# A Digital Screening System for Alzheimer Disease Based on a Neuropsychological Test and a Convolutional Neural Network: System Development and Validation

**DOI:** 10.2196/31106

**Published:** 2022-03-09

**Authors:** Wen-Ting Cheah, Jwu-Jia Hwang, Sheng-Yi Hong, Li-Chen Fu, Yu-Ling Chang, Ta-Fu Chen, I-An Chen, Chun-Chen Chou

**Affiliations:** 1 Department of Computer Science and Information Engineering National Taiwan University Taipei Taiwan; 2 Department of Psychology National Taiwan University Taipei Taiwan; 3 Department of Neurology National Taiwan University Hospital Taipei Taiwan; 4 Taipei City Zhishan Senior Home Taipei Taiwan

**Keywords:** Alzheimer disease, mild cognitive impairment, screening system, convolutional neural network, Rey-Osterrieth Complex Figure

## Abstract

**Background:**

Alzheimer disease (AD) and other types of dementia are now considered one of the world’s most pressing health problems for aging people worldwide. It was the seventh-leading cause of death, globally, in 2019. With a growing number of patients with dementia and increasing costs for treatment and care, early detection of the disease at the stage of mild cognitive impairment (MCI) will prevent the rapid progression of dementia. In addition to reducing the physical and psychological stress of patients’ caregivers in the long term, it will also improve the everyday quality of life of patients.

**Objective:**

The aim of this study was to design a digital screening system to discriminate between patients with MCI and AD and healthy controls (HCs), based on the Rey-Osterrieth Complex Figure (ROCF) neuropsychological test.

**Methods:**

The study took place at National Taiwan University between 2018 and 2019. In order to develop the system, pretraining was performed using, and features were extracted from, an open sketch data set using a data-driven deep learning approach through a convolutional neural network. Later, the learned features were transferred to our collected data set to further train the classifier. The first data set was collected using pen and paper for the traditional method. The second data set used a tablet and smart pen for data collection. The system’s performance was then evaluated using the data sets.

**Results:**

The performance of the designed system when using the data set that was collected using the traditional pen and paper method resulted in a mean area under the receiver operating characteristic curve (AUROC) of 0.913 (SD 0.004) when distinguishing between patients with MCI and HCs. On the other hand, when discriminating between patients with AD and HCs, the mean AUROC was 0.950 (SD 0.003) when using the data set that was collected using the digitalized method.

**Conclusions:**

The automatic ROCF test scoring system that we designed showed satisfying results for differentiating between patients with AD and MCI and HCs. Comparatively, our proposed network architecture provided better performance than our previous work, which did not include data augmentation and dropout techniques. In addition, it also performed better than other existing network architectures, such as AlexNet and Sketch-a-Net, with transfer learning techniques. The proposed system can be incorporated with other tests to assist clinicians in the early diagnosis of AD and to reduce the physical and mental burden on patients’ family and friends.

## Introduction

### Background

According to the latest report from Alzheimer’s Disease International [1], the number of people with dementia worldwide will increase from 50 million in 2019 to 152 million by 2050, and the global annual cost of dementia is estimated to increase from US $1 trillion in 2019 to US $2 trillion in 2030. Dementia is also the seventh-leading cause of death in the world [2]. These numbers continue to grow year by year, and the risk of developing dementia grows significantly with increasing age. Therefore, as more and more countries’ aging populations increase, there is an urgent need to put more effort into research related to this issue, since there is no cure for AD and the existing treatment is to extend the period of rapid progression of the disease.

AD is the most common etiology associated with dementia, and it accounts for approximately 60% to 70% of all dementia cases [3]. AD caused by the destruction and death of neurons in the brain is a syndrome related to ongoing decline in cognitive function in domains such as memory, visuospatial processing, language, and executive function; this decline results in impairment in carrying out the instrumental and basic activities of daily living [4].

**Figure 1:**
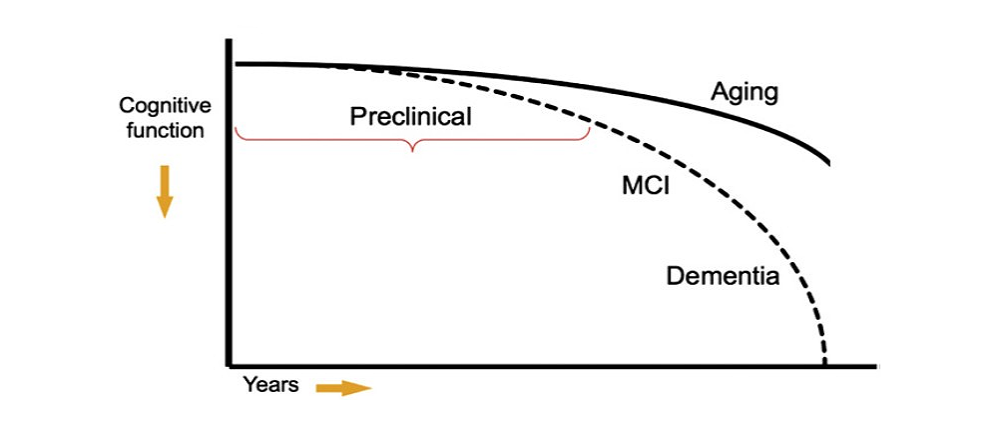
The continuum of Alzheimer disease [5]. MCI: mild cognitive impairment.

MCI is a transitional state between normal aging and dementia, in which a patient’s cognitive function undergoes mild but perceptible decline, as shown in Figure 1 [5]. Such degradation of cognitive function occurs more quickly than in normal aging, but unlike in AD, it does not affect the patient’s ability to handle daily activities. According to the updated American Academy of Neurology guideline on MCI [6], about 14.9% of patients with MCI older than 65 years of age developed dementia at a 2-year follow-up. In clinical trials involving patients with MCI who had memory loss, most of them who progressed to having dementia had AD.

Currently, the diagnoses of the MCI and AD are based on the clinical judgment of doctors according to the symptoms, medical reports, and medical history from the individual, family members, friends, or caregivers. Additionally, a series of cognitive tests and neuropsychological assessments, such as the Mini-Mental State Examination (MMSE) [7] and the Clinical Dementia Rating scale [8], are essential to evaluate the individual’s cognitive function. Furthermore, biomarker measurements that include cerebrospinal fluid testing and neuroimaging, such as structural magnetic resonance imaging (MRI) and positron emission tomography (PET), are also used to aid in diagnosis [9].

Several challenges need to be addressed to propose a screening system for the early detection of AD. One of the challenges is that the characteristics or signs of the early stage of the disease may not be obvious [10]. Moreover, the high cost of manual feature extraction needs to be avoided. However, meaningful feature representation has to be determined for building a screening model for the disease. As a screening tool, the efficiency of the overall screening process is another issue that needs to be considered. In this work, we proposed a digital screening system to reduce the burden on clinicians.

### Purpose

The aim of this research was to propose a data-driven convolutional neural network (CNN) architecture through transfer learning and deep learning methods to discriminate between patients with AD or MCI and healthy controls (HCs). The designed CNN architecture was developed for a Rey-Osterrieth Complex Figure (ROCF) test system that automatically calculates scores to assist diagnosis. The purpose of the proposed system is to prevent late diagnosis of AD among older adults. Nevertheless, the proposed system will also reduce the manual workload for clinicians and diagnostic costs.

### Related Work

#### Overview

When AD and other types of dementia collectively became one of the primary public health concerns worldwide, many different types of research studies began to develop diagnostic tools to accurately classify individuals as having AD or MCI or as cognitively unimpaired individuals, also known as HCs. These studies can be categorized into two main types: neuroimaging studies and neuropsychological test studies.

#### Neuroimaging Studies

AD is a neurodegenerative disease, and the most remarkable brain changes appear to occur in the hippocampal formation and the entorhinal cortex, which are critical brain structures related to memory function. MRI is commonly used to measure the structural atrophy of the hippocampus and entorhinal cortex. Compared with cognitively unimpaired older adults and individuals with MCI, patients with AD have a smaller-sized hippocampus and entorhinal cortex [11]. Functional MRI provides information on the flow of oxygenated blood in the brain to detect higher brain cell activity by higher blood flow; it can be used to record the activation patterns of neural networks in the hippocampus when the participant is performing memory tests [12]. Furthermore, with the injection of a radioactive contrast agent into the human body, a PET scan can be used to obtain information on glucose metabolism and the brain’s neurotransmitter activity.

With the help of multiscale feature extraction from baseline local hippocampus MRI data, Hu et al [13] adopted support vector machine (SVM) learning models to distinguish between patients with MCI that converted to AD and patients with MCI that did not convert to AD, and to distinguish between patients with AD and HCs. Challis et al [14] applied functional MRI scans and Bayesian Gaussian process logistic regression models to distinguish between HCs and patients with amnestic MCI, and between patients with amnestic MCI and those with AD. Li et al [15] used fusion information from MRI and PET scans for feature selection, processed the selected features through restricted Boltzmann machines to obtain the learned features, and applied the learned features to an SVM model for the classification of the different stages of AD. However, neuroimaging is not cheap. Moreover, patients who experience claustrophobia cannot undergo scanning by the machine because patients need to lie motionless inside the closed shell of the machine. Furthermore, patients with metallic implants, such as pacemakers, cannot undergo MRI due to the magnetic and radiofrequency fields generated during imaging. In addition, patients will be exposed to radiation while undergoing a PET scan.

#### Neuropsychological Test Studies

Neuropsychological assessments employing specifically designed tests are important for evaluating the brain dysfunction’s behavioral and functional expression [16]. A neuropsychological test is typically administered to a participant by an examiner or neuropsychologist in a quiet environment. The purpose of the assessment is to gather the participant’s cognitive and behavioral performance information. The MMSE is a widely used screening test for evaluating the cognitive status of older adults. However, it has limited utility in distinguishing between the patients with MCI and people in a standard aging group [17].

Drawing tests are widely used to assess constructional abilities, where the patient is asked to copy a complex figure and then recall and replicate the figure from memory. The Clock-Drawing Test (CDT) is a simple tool for screening people with dementia. It requires participants to draw the clock correctly using appropriate abilities, such as understanding language, planning, visualizing orientations, and executing the appropriate movement. However, people with dementia will not draw correctly due to impaired cognitive abilities, such as visual constructional processing, memory function, semantic knowledge retrieval, or executive function. Prange and Sonntag [18] proposed a digital CDT by implementing the Mendez scoring system [19] and creating a hierarchy of error categories to model the characteristics of CDT. Nevertheless, according to a survey report [20], many researchers using the CDT cannot significantly distinguish between patients with MCI and cognitively unimpaired participants, and the sensitivity and specificity have also been less satisfactory in most studies.

The ROCF test is widely used to assess visuospatial constructional capabilities and visual memory function [21]. It is a score-based neuropsychological assessment tool that assesses the individual’s visual memory by testing their ability to draw a complex figure by copying, immediate recall, and delayed recall from memory. The ROCF test was first constructed by Rey [22], and it was then standardized by Osterrieth [23]. Miller et al [24] showed that combining the ROCF test with the MMSE can enhance the performance of the detection of individuals with MCI. Salvadori et al [25] evaluated the ROCF test using the Boston Qualitative Scoring System (BQSS) [26] in order to distinguish vascular MCI from degenerative MCI. There are several different scoring systems for quantifying the performance of the ROCF test, for example, the Rey-Osterrieth 36-point scoring system [27], the Developmental Scoring System [28], and the BQSS.

Nevertheless, the current scoring system of the ROCF test is labor intensive and needs to be performed by trained experts, due to the complexity of the scoring criterion. However, cognitive impairment in individuals with MCI is often subtle. It appears to be more challenging to distinguish between patients with MCI and HCs than between patients with AD and HCs, as the current manual scoring system may also have a limited ability to detect subtle differences between individuals with MCI and HCs.

## Methods

### System Overview

**Figure 2:**
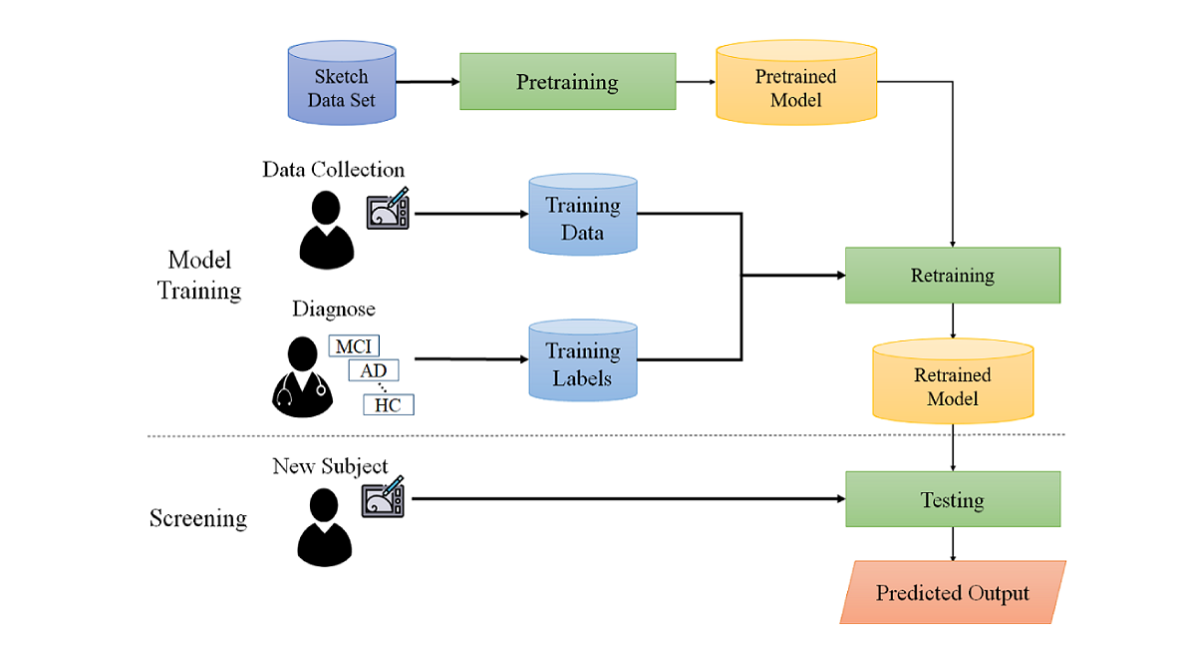
System overview. AD: Alzheimer disease; HC: healthy control; MCI: mild cognitive impairment.

The proposed approach was partitioned conceptually into two portions, namely model training and screening, as depicted in Figure 2. The model training portion mainly included three modules: data collection, pretraining, and retraining. First, according to the standardized assessment protocol, participants had to be classified as individuals with MCI or HCs by an experienced doctor and neuropsychologist. Therefore, the diagnosis results were used to train the classification model as the ground truth. Second, we collected the ROCF test drawings from all participants, and a large, open, sketch data set was used for pretraining our proposed screening system. Third, the screening model was implemented by applying the pretrained model to the collected data. Finally, we used the retraining model to discriminate participants. The screening portion used the system to classify new participants by differentiating cognitively unimpaired individuals from patients with AD or MCI. The following sections discuss the detailed implementation of each part in more detail.

### Screening System

#### Neuropsychological Test Selection

Neuropsychological test selection was based on whether it could be performed in a clinical setting and whether it had been used in related AD and MCI studies. The ROCF test is a neuropsychological test that has been adopted to assess various cognitive functions, such as visuospatial abilities, visual episodic memory, organization skills, attention, and visuomotor coordination [29]. Visual memory impairment is an early sign of AD [30], and some studies [31,32] have shown that the ROCF test can identify patients with MCI, patients with mild AD, and HCs.

The ROCF does not resemble any existing object; it combines many shapes that include lines, circles, rectangles, triangles, crosses, diamonds, and more. There are three trials during an ROCF test: copy, immediate recall, and delayed recall. Cognitive functions such as attention, visuospatial processing, and visuomotor coordination are required for copying the complicated geometrical figures successfully. The immediate recall and the delayed recall are used to assess the participant’s ability to retrieve learned information from memory incidentally.

#### Data Collection Procedure

##### Overview

First, participants were invited to participate in the study according to ethical approval from the Institutional Review Board (IRB) of the National Taiwan University Hospital (NTUH; see Ethics Approval section for details), and written informed consent was received from each of them. Each participant was asked to sit at a table with pen and paper or with a Cintiq 16 tablet (Wacom) and Pro Pen 2 (Wacom) [33]. Next, the participant was asked to write his or her name or draw some shapes on the digital device using the smart pen in order to become familiar with the devices. After that, the participant was informed about the process of the ROCF test during three trials: the copy trial, the immediate recall trial, and the delayed recall trial.

##### Copy Trial

The participant was shown the ROCF and asked to duplicate the complicated geometrical figure as close as possible to the original figure. The participant was informed that there was no time limit for copying the figure. After the copy stage was finished, both the original ROCF and the copied figure that was drawn by the participant were removed from sight. Furthermore, the participant was not notified that the figure would need to be drawn again in the subsequent trials.

##### Immediate Recall Trial

After a short delay, the participant was asked to draw the complicated geometrical figure from memory with as much detail as possible. The participant was informed that there was no time limit. When the immediate recall drawing was finished, the drawn figure was moved away from the participant’s sight.

##### Delayed Recall Trial

After a 20- to 30-minute delay, the participant was asked to redraw the complicated geometrical figure from memory. The participant was informed that there was no time limit.

### Data Preparation

#### Overview

Two different data sets were used to evaluate our proposed AD screening system; they were gathered according to ethical approval from the IRB of the NTUH (see Ethics Approval section). In line with IRB ethical approval, older adults in Taiwan were invited, and their written informed consent was obtained. Participants with a past or current history of the following conditions were excluded from this study:

1. Nonneurodegenerative problems that might affect brain function, such as stroke, epilepsy, and moderate or severe head injury.
2. Severe psychiatric illness, such as depression and autism.
3. Drug abuse.
4. Blindness or severe hearing impairment that would result in participants not being able to take the ROCF neuropsychological test.

The details of both data sets are described in the following sections.

#### NTUH_ROCF Data Set

This study data set included a total of 118 participants: 59 (50.0%) participants with MCI and 59 (50.0%) HCs. The NTUH_ROCF data set was collected using pen and paper through the data collection procedure described above. All participants underwent a comprehensive neuropsychological assessment, including measurements from five cognitive domains: attention, executive function, visuospatial function, memory, and language. An expert from our team evaluated the assessments; the criteria of classifying patients with MCI was based on the approaches proposed by Jak et al [34].

Table 1 shows the demographic information of the older adult participants, including gender, age, years of education (minimum 6 years), and MMSE scores for the MCI and HC groups. Participants were asked to draw the ROCF pictures during the copy trial, the 3-minute delayed trial (ie, immediate recall), and the 30-minute delayed trial (ie, delayed recall).

**Table 1:** Demographic information from the NTUH_ROCF^a^ data set.

Characteristic			Participants with mild cognitive impairment (n=59)		Healthy controls (n=59)		
**Gender, n (%)**							
	Female	31 (53)		33 (56)			
	Male	28 (47)		26 (44)			
Age (years), mean (SD)			67.51 (6.30)		62.58 (5.89)		
Education (years), mean (SD)			13.12 (3.20)		15.05 (2.82)		
MMSE^b^ score, mean (SD)			27.81 (2.10)		29.18 (0.96)		

^a^NTUH_ROCF: National Taiwan University Hospital_Rey-Osterrieth Complex Figure.

^b^MMSE: Mini-Mental State Examination; total scores range from 0 (all answers are incorrect) to 30 (all answers are correct).

#### NTUH_D-ROCF Data Set

This study data set included a total of 60 participants: 30 (50%) participants with AD and 30 (50%) HCs. Patients with AD were recruited from NTUH, and the NTUH_D-ROCF data set (where “D” represents Alzheimer disease) was collected using the graphics tablet and smart pen (Cintiq 16 and Pro Pen 2; Wacom) to evaluate the automation approach’s performance. Disease diagnoses from a board-certified neurologist and a board-certified clinical neuropsychologist were used as the ground truth for training the system. The demographic information from the participants is summarized in Table 2. Participants were asked to draw the ROCF pictures during the copy trial, the immediate recall trial, and the 10-minute delayed recall trial.

**Table 2:** Demographic information from the NTUH_D-ROCF^a^ data set.

Characteristic		Participants with Alzheimer disease (n=30)		Healthy controls (n=30)
**Gender, n (%)**				
	Female	20 (67)	19 (63)	
	Male	10 (33)	11 (37)	
Age (years), mean (SD)		77.67 (6.96)		73.40 (7.24)
Education (years), mean (SD)		11.83 (3.55)		15.03 (2.66)
MMSE^b^ score, mean (SD)		21.33 (2.80)		28.50 (1.55)

^a^NTUH_D-ROCF: National Taiwan University Hospital_Alzheimer Disease_Rey-Osterrieth Complex Figure.

^b^MMSE: Mini-Mental State Examination; total scores range from 0 (all answers are incorrect) to 30 (all answers are correct).

#### Designed Architecture of the Neural Network

##### Overview

**Figure 3:**
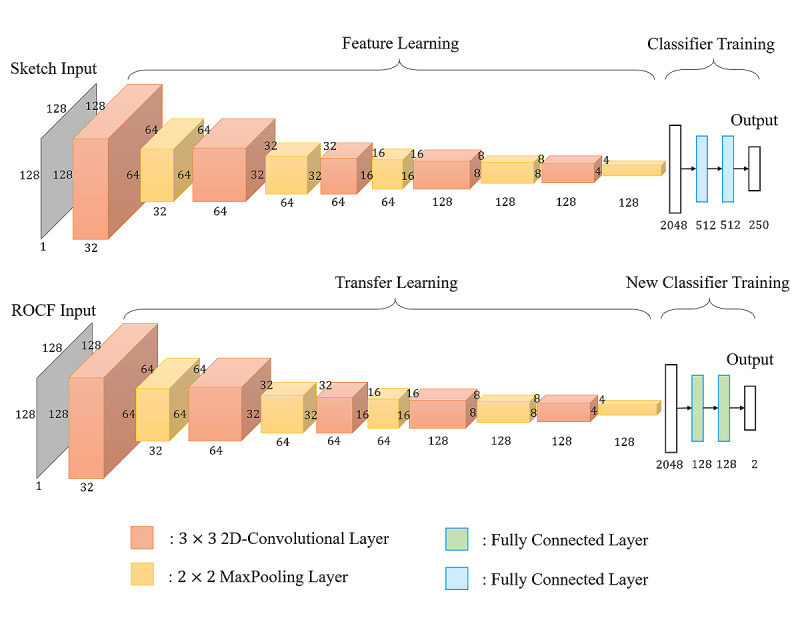
The network structure of the screening system for Alzheimer disease. ROCF: Rey-Osterrieth Complex Figure.

Training a deep CNN from scratch is a time-consuming task and usually requires a large amount of data to achieve the goal of generalization. Generally, it is hard for researchers to collect enough labeled images for each specific task. According to research by Yosinski et al [35], the transfer learning technique applied to deep neural networks could achieve surprising results. They found that initializing the weights of a network by transferring features from almost any number of layers of a pretrained network can retain the generalization ability, even fine-tuning the weights according to the target data set. It inspired us to use the TU (Technical University)-Berlin sketch data set [36] to pretrain our neural network. The data set consists of 250 different object categories, such as animal, insect, plant, food, furniture, transportation, and instrument, where each category contains 80 sketch images. The data set contains a total of 20,000 hand-drawn sketches. We used that data set because it is large and similar to our collected data, in that both sets of images are sketched and contain the shapes of circles, squares, and lines. The learned weights or pretrained models were then transferred to the target screening engine rather than training the target neural network from scratch. The network structure of our proposed screening system is depicted in Figure 3.

##### Pretraining Engine

Inspired by the neural network architecture described in Yu et al [37], we further designed a low-cost neural network for pretraining the sketch data set, as demonstrated in the upper part of Figure 3. The input was the image *I*∈*R^h^*^×^*^w^*^×^*^c^*, where *h* and *w* stand for the height and width of the image, and *c* is the number of channels of the image. The output comprised the probabilities of belonging to the corresponding 250 categories, and the highest probability indicated the predicted output label of the sketch image. In other words, features were extracted based on CNN architecture to recognize the hand-drawn sketches across numerous object categories. A series of convolutional layers and their following pooling layers acted as the feature extraction, while fully connected (FC) layers were used for further classification.

The pretraining network adopted five 3×3 convolutional layers with a stride of 1 pixel. The stride was set to 1 to keep as much information as possible through the convolution operation. In addition, each convolutional layer was followed by a 2×2 max pooling layer using a stride of 2 pixels. The convolutional function used in each convolutional layer is represented as follows:

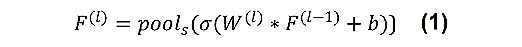



where *F*^(^*^l^*^–1)^ indicates the input feature map to the *l*-th layer, *W* is the weight matrix to be applied to the input feature map, *b* is the bias vector, the operator * is the convolution operation, *σ* is the nonlinear activation function, *pool* is a subsampling operation, and *s* represents the pooling size of the filter that usually covers an *s*×*s* square region.

The feature representations were then flattened into a 2048-dimension vector and connected to two FC hidden layers, each with 512 neurons. A rectified linear unit (ReLU) [38] function, as shown in equation 2 below, was used as the activation function of the convolutional layers and the two FC hidden layers, while the softmax function, as shown in equation 3 below, was applied to the output layer to compute the prediction probability for each class. Finally, dropout [39] was adopted after the flattened layer and two FC hidden layers with a dropout rate of 0.5. The dropout technique was used to prevent overfitting of the training data, which reduced the number of active neurons during training by dropping 50% of the neurons.

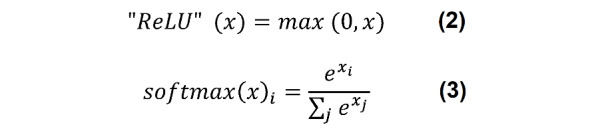



**Figure 4:**
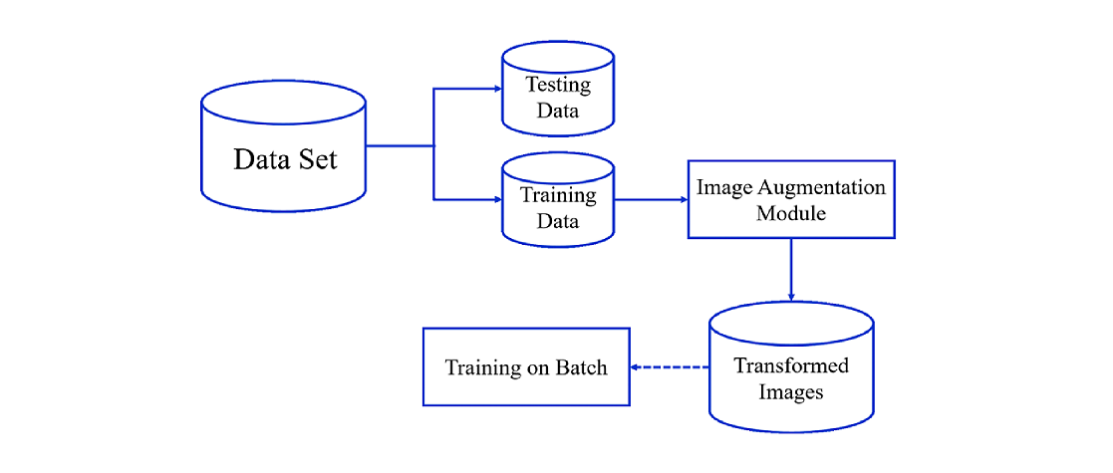
The real-time process of data augmentation.

A data augmentation technique was used to increase the diversity of sketches per category for classification. Furthermore, it increased the number of training samples through several random transformations on the image, such as vertical shift, horizontal shift, rotation, and flip, in order to train the model with a greater range of various augmented data. This technique lets the model constantly train on new, slightly modified versions of the input data, which enables the model to learn more robust features and increases the generalization of the model. Thus, the shift and rotation transformations of data augmentation were adopted in the training process, and the transformations were then applied in real time as batches were passed into training in this work, as shown in Figure 4.

Initially, the sketch data set was separated into training and testing data, and the data augmentation technique was only applied to the training data. The original batch of sketch images was then fed into the image augmentation module to apply a series of random transformations to each image in the batch. Next, the sketches from the training data were randomly shifted horizontally or vertically with a 0.1 fraction of total width or height and randomly rotated in the range of 0.1 degrees. Finally, the new and randomly transformed batch was used for training the CNN, while the original data were not used for training. In other words, the image augmentation module randomly transformed the original images and returned only the new transformed images.

The cross-entropy loss function was applied to calculate the model loss through the training data. We obtained the loss value for later optimization by comparing the model’s predictions with the ground truth. The probability 
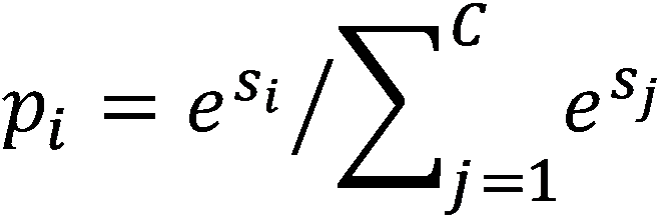
 denotes the prediction result of *i*-th class of a sample, where *s* is the output score of the model, *s_i_* is the *i*-th element of vector *s*, and *C* is the total number of the classes. Set *y* = [*y*_1_,...,*y_M_*,...,*y_C_*], where *y_M_* = 1 and *y_i_* = 0(if *i* ≠ *M*) to indicate that the *M*-th class is the ground truth. Then, the cross-entropy loss function *L* is represented as follows:

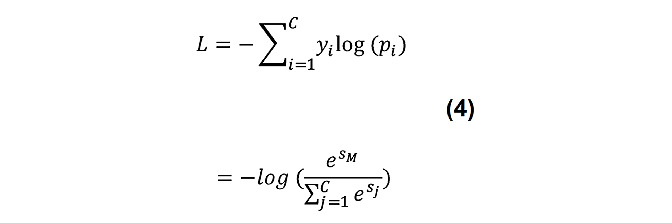



Lastly, an Adam optimizer [40] with a learning rate of 0.0001 was used to adjust the trainable parameters to reduce the model loss for each batch. The Adam optimizer combines two methods: AdaGrad (adaptive gradient algorithm) [41], which deals with sparse gradients very well, and RMSProp (root mean square propagation), which does well with online and nonstationary settings.

##### Retraining Engine

Given the image as the input *I*∈*R^h^*^×^*^w^*^×^*^c^*, where *h* and *w* stand for the height and width of the image and *c* is the number of channels of the image, which implicitly contains the necessary information for building the screening model of AD, the output is the score or probability of having AD or MCI. A CNN was used to determine the score, and features were learned automatically rather than handcrafted. We formulated the score of having AD or MCI with a function of the image drawn by the participant, as shown in the following equation:

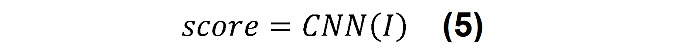



where *I* represents the image drawn by the participant, the output of the model *score* is a 2D vector, and each dimension is a scalar value between 0 and 1, which indicates the possibility of having AD or MCI or of being an HC, respectively.

The architecture of the retraining engine is shown in the bottom part of Figure 3. The convolutional base of the retraining engine was leveraged from the convolutional base of the pretraining model by using the TU-Berlin sketch data set. The convolutional base layers were frozen, consisting of five convolutional layers with a filter size of 3×3 and a stride of 1 pixel; a 2×2 max pooling layer follows each convolutional layer with a stride of 2 pixels. A new classifier was implemented for further distinguishing the image drawn by the participant. The feature representations were then flattened into a 2048-dimension vector and connected to two FC layers, each with 128 neurons. Every node of the FC layer applied the ReLU [38] activation function (equation 2). The probability or score of having AD or MCI was calculated by applying the softmax function (equation 3). The dropout technique was also applied after the flattened layer with a dropout rate of 0.5.

The same data augmentation technique applied in the pretraining engine was also implemented in the retraining engine. The ROCF training data were randomly shifted horizontally or vertically with a 0.05 fraction of total width or height and randomly rotated in the range of 0.1 degrees. As mentioned before, the real-time data augmentation technique implemented in the screening engine was similar to that implemented in the pretraining engine.

The corresponding ground truth label for the output is as follows: 0 indicates that the participant is healthy, while 1 indicates that the participant has AD or MCI. The loss function is defined as the cross-entropy sum between the predicted output and the ground truth as follows:

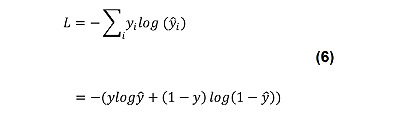



where *L* is the loss function, *y_i_* is the ground truth of class *i*, and *y* and *ŷ* are the truth label and output of the screening engine, respectively. In addition, the Adam optimizer with a learning rate of 0.0001 was adopted for training the retraining engine, and the batch size was 16 for the training process.

### Ethics Approval

Ethical approval was obtained from the IRB (No. NTUH 201802091RIND) of the NTUH.

## Results

### Performance Metrics

The performance of the system was measured using four metrics: sensitivity, specificity, accuracy, and area under the receiver operating characteristic curve (AUROC). Sensitivity represents the proportion of actual patients with AD or MCI who are identified correctly. Specificity denotes the proportion of people who are genuinely healthy older adults who are identified correctly. Accuracy indicates the ratio of correctly classified patients with MCI or AD and cognitively unimpaired older adults to total participants. The receiver operating characteristic (ROC) curve illustrates the relationship between sensitivity and specificity for a given classification model and several given thresholds. If the ROC curve is almost a straight line through the diagonal, it indicates poor performance. When comparing different classification models, the ROC curve of each model can be drawn, and the AUROC is used as an indicator to illustrate the model’s performance. Equations for calculating sensitivity and specificity are as follows:

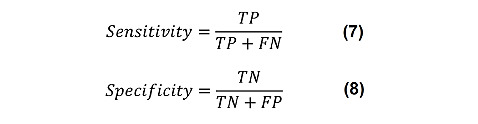



where *TP* (true positive) and *TN* (true negative) denote the number of correct classifications, and where *FP* (false positive) and *FN* (false negative) denote the number of the incorrect classifications.

### Evaluation Procedure

A series of experiments were conducted to examine the efficiency of our proposed screening engine. First, the images were resized to 128×128×1, and the data were randomly shuffled to ensure that they were thoroughly mixed. Next, training and testing were executed on a GeForce GTX 1080 Ti GPU (NVIDIA) to evaluate the performance of the implemented classifier through a 10-fold cross-validation procedure. The data set was randomly shuffled to 10 subsets, which were used as testing data in turn, and the other nine subsets were used as training data for each fold test. The 10-fold cross-validation was repeated five times, and each performance score was recorded.

### Evaluation of the NTUH_ROCF Data Set

#### Comparison of Different ROCF Trials

The performance of the copy, immediate recall, and delayed recall trials were calculated separately, and the results are listed in Table 3. The performance of the copy trial had a mean sensitivity of 0.668 (SD0.015), a mean specificity of 0.536 (SD0.026), a mean accuracy of 0.602 (SD0.009), and a mean AUROC of 0.672 (SD0.004). The results of the copy trial indicate that it was not easy to distinguish whether a participant had MCI or was an HC; this might be the case because both patients with MCI and HCs still have adequate attention and visuospatial processing ability, allowing them to duplicate the complex geometrical figure during the copy trial. On the contrary, the delayed recall trial had the best classification capability in differentiating participants with MCI from HCs, with a mean sensitivity of 0.847 (SD0.017), a mean specificity of 0.905 (SD0.009), a mean accuracy of 0.876 (SD0.010), and a mean AUROC of 0.913 (SD0.004). The performance of the immediate recall trial had a mean sensitivity of 0.736 (SD0.028), a mean specificity of 0.885 (SD0.014), a mean accuracy of 0.810 (SD0.015), and a mean AUROC of 0.871 (SD0.008). Compared with cognitively unimpaired older adults, the patients with MCI may have had problems recalling the figure from memory after some time.

**Table 3:** Performance of three ROCF^a^ trials using the NTUH_ROCF^b^ data set.

Metric	Copy trial, mean (SD)	Immediate recall trial, mean (SD)	Delayed recall trial, mean (SD)
Sensitivity	0.668 (0.015)	0.736 (0.028)	0.847(0.017)
Specificity	0.536 (0.026)	0.885 (0.014)	0.905 (0.009)
Accuracy	0.602 (0.009)	0.810 (0.015)	0.876 (0.010)
AUROC^c^	0.672 (0.004)	0.871 (0.008)	0.913 (0.004)

^a^ROCF: Rey-Osterrieth Complex Figure.

^b^NTUH_ROCF: National Taiwan University Hospital_Rey-Osterrieth Complex Figure.

^c^AUROC: area under the receiver operating characteristic curve.

#### Performance of the Proposed Screening System for Classifying Participants With MCI Versus Healthy Controls

**Figure 5:**
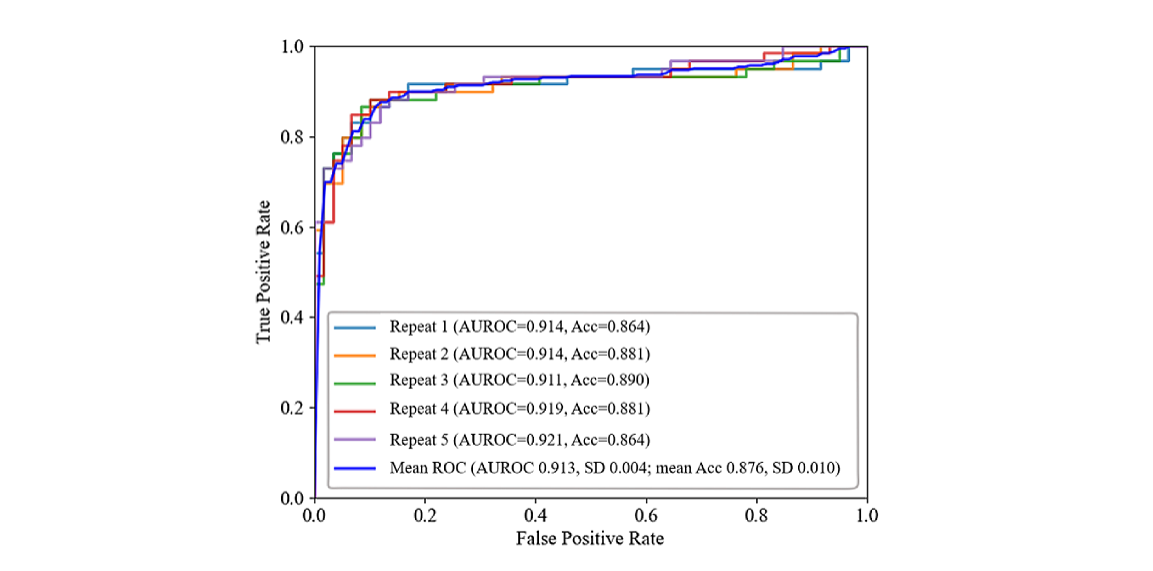
Receiver operating characteristic (ROC) curves of the proposed screening engine after five repeats of 10-fold cross-validation using the NTUH_ROCF data set. Acc: accuracy; AUROC: area under the receiver operating characteristic curve; NTUH_ROCF: National Taiwan University Hospital_Rey-Osterrieth Complex Figure.

In this experiment, the performance of the proposed architecture of the screening engine for distinguishing between the complex figures drawn by participants with MCI and HCs was evaluated using the images drawn by the participants during the delayed recall trial. Initially, the TU-Berlin sketch data set was used to pretrain the neural network; the learned feature representations were then leveraged for our ROCF data set for further training. Figure 5 shows the mean (SD) of AUROC and accuracy for each repeat of the 10-fold cross-validation and the mean (SD) of these five repeats. The performance of our model achieved a mean AUROC of 0.913 (SD 0.004), while the mean accuracy of the five repeats of 10-fold cross-validation was 0.876 (SD 0.010).

### Evaluation of the NTUH_D-ROCF Data Set

#### Comparison of Different ROCF Trials

Different ROCF trials were evaluated individually, and their performance results are shown in Table 4. The performance of the immediate recall trial and the 10-minute delayed recall trial were similar in distinguishing between participants with AD and HCs. The performance of delayed recall had a mean sensitivity of 0.820 (SD0.038), a mean specificity of 0.953 (SD 0.018), a mean accuracy of 0.887 (SD 0.016), and a mean AUROC of 0.940 (SD 0.006). The performance of immediate recall had a mean sensitivity of 0.827 (SD 0.015), a mean specificity of 0.947 (SD 0.018), a mean accuracy of 0.887 (SD 0.012), and a mean AUROC of 0.950 (SD 0.003). The results showed that the immediate recall trial had the best performance, followed by the 10-minute delayed recall trial, while both could be used to distinguish between participants with AD and HCs. The patients with AD may have had problems recalling the complex figure from memory during the immediate recall trial and the 10-minute delayed recall trial, as compared to HCs. On the other hand, compared with the immediate recall trial or the 10-minute delayed recall trial, the performance of the copy trial was less discriminative; this trial had a mean sensitivity of 0.627 (SD 0.028), a mean specificity of 0.900 (SD 0.033), a mean accuracy of 0.763 (SD 0.016), and a mean AUROC of 0.762 (SD 0.018).

**Table 4:** Performance of three ROCF^a^ trials using the NTUH_D-ROCF^b^ data set.

Metric	Copy trial, mean (SD)	Immediate recall trial, mean (SD)	Delayed recall trial, mean (SD)
Sensitivity	0.627 (0.028)	0.827 (0.015)	0.820 (0.038)
Specificity	0.900 (0.033)	0.947 (0.018)	0.953 (0.018)
Accuracy	0.763 (0.016)	0.887 (0.012)	0.887 (0.016)
AUROC^c^	0.762 (0.018)	0.950 (0.003)	0.940 (0.006)

^a^ROCF: Rey-Osterrieth Complex Figure.

^b^NTUH_D-ROCF: National Taiwan University Hospital_Alzheimer Disease_Rey-Osterrieth Complex Figure.

^c^AUROC: area under the receiver operating characteristic curve.

#### Performance of the Proposed Screening System for Classifying Participants With AD Versus Healthy Controls

**Figure 6:**
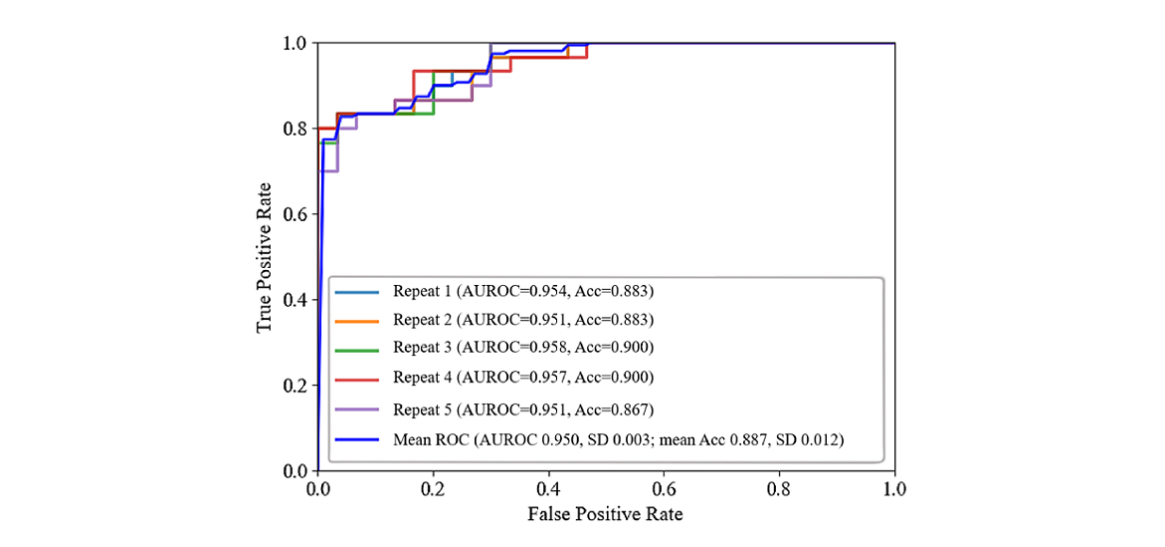
Receiver operating characteristic (ROC) curves of the proposed screening engine after five repeats of 10-fold cross-validation using the NTUH_D-ROCF data set. Acc: accuracy; AUROC: area under the receiver operating characteristic curve; NTUH_D-ROCF: National Taiwan University Hospital_Alzheimer Disease_Rey-Osterrieth Complex Figure.

The performance of the proposed architecture of the screening engine to distinguish between the abstract and complex figures drawn by participants with AD and HCs was conducted using the images collected from the immediate recall trial. First, the TU-Berlin sketch data set was also used to pretrain the neural network; the feature representations learned by the pretrained neural network were then fine-tuned and leveraged for our ROCF data set for further training. As a result, the performance of our model achieved a mean AUROC of 0.950 (SD 0.003), while the mean accuracy of the five repeats of 10-fold cross-validation was 0.887 (SD 0.012), as shown in Figure 6.

### Effectiveness of the Dropout and Data Augmentation Techniques

An experiment verifying the performance of the designed system after applying the data augmentation and dropout techniques was conducted for the data collected in the delayed recall trial. The data augmentation method was adopted when training the neural network concurrently; only the training data, instead of testing data, were augmented. The performance of the designed system after applying both techniques was better, with a mean sensitivity of 0.847 (SD 0.017), a mean specificity of 0.905 (SD 0.009), a mean accuracy of 0.876 (SD 0.010), and a mean AUROC of 0.913 (SD 0.004). When the techniques were not used, the system had a mean sensitivity of 0.824 (SD 0.019), a mean specificity of 0.898 (SD0.024), a mean accuracy of 0.861 (SD 0.001), and a mean AUROC of 0.893 (SD 0.012), as seen in Table 5. When applying data augmentation and dropout techniques, most studies on image classification obtain better results. Data augmentation techniques could extend the diversity of the training data, and dropout techniques could avoid coadaptation of the model by randomly disabling neurons with probability during the training process. The results showed that with the data augmentation and dropout techniques, the system performed better, according to the results provided in Table 5. Therefore, integrating them into the model provides better results. Consequently, to obtain better performance, both technologies were adopted in our model. The higher the sensitivity, specificity, accuracy, and AUROC values, the better the performance was. However, these numbers do not explain the system’s reliability, sustainability, and consistency. That will be a different concern to address, which is out of the scope of this research.

**Table 5:** Effects of data augmentation and dropout techniques applied to the NTUH_ROCF^a^ data set.

Metric	Delayed recall trial, mean (SD)	
	Without data augmentation and dropout techniques	With data augmentation and dropout techniques
Sensitivity	0.824 (0.019)	0.847 (0.017)
Specificity	0.898 (0.024)	0.905 (0.009)
Accuracy	0.861 (0.011)	0.876 (0.010)
AUROC^b^	0.893 (0.012)	0.913 (0.004)

^a^NTUH_ROCF: National Taiwan University Hospital_Rey-Osterrieth Complex Figure.

^b^AUROC: area under the receiver operating characteristic curve.

### Comparison of Different Network Architectures

From the images drawn by participants in the delayed recall trial, the performances of the different architectures of the neural network classifier were studied. Additionally, the total number of parameters and the time to complete 1-fold training were listed for comparison for different classifiers. The different neural network architectures included AlexNet [42]; Sketch-a-Net [37]; our previous work, a convolutional autoencoder neural network [43]; and the proposed network architectures mentioned in this study. As a result, the architecture of our proposed framework in this study achieved better performance than the architecture of AlexNet, Sketch-a-Net, and our previous work, as shown in Table 6.

**Table 6:** Performance of different network architectures applied to the NTUH_ROCF^a^ data set.

Metric	AlexNet	Sketch-a-Net	Our system	
			Without data augmentation and dropout techniques	With data augmentation and dropout techniques
Sensitivity, mean (SD)	0.698 (0.039)	0.671 (0.047)	0.756 (0.033)	0.847 (0.017)
Specificity, mean (SD)	0.790 (0.046)	0.820 (0.054)	0.864 (0.017)	0.905 (0.009)
Accuracy, mean (SD)	0.744 (0.034)	0.746 (0.019)	0.810 (0.020)	0.876 (0.010)
AUROC,^b^ mean (SD)	0.814 (0.021)	0.819 (0.009)	0.851 (0.020)	0.913 (0.004)
Total parameters (×10^6^), n	46.73	8.38	0.40	0.56
Time required to complete 1-fold training, minutes	10	29	9	2

^a^NTUH_ROCF: National Taiwan University Hospital_Rey-Osterrieth Complex Figure.

^b^AUROC: area under the receiver operating characteristic curve.

**Figure 7:**
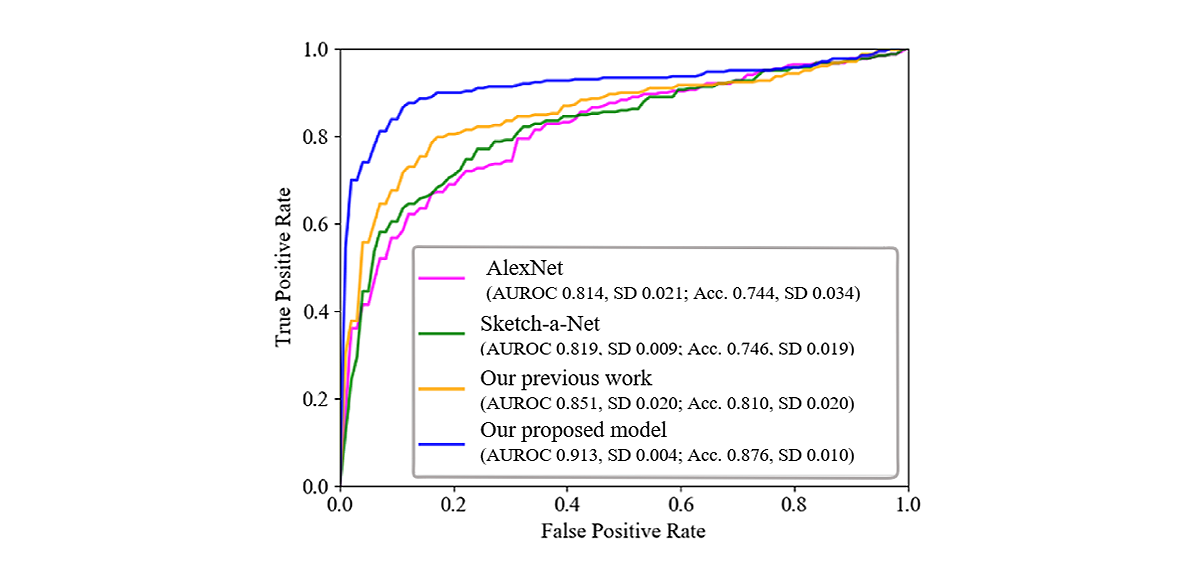
Receiver operating characteristic curves of the different network architectures using the NTUH_ROCF data set. Acc: accuracy; AUROC: area under the receiver operating characteristic curve; NTUH_ROCF: National Taiwan University Hospital_Rey-Osterrieth Complex Figure.

The sensitivity, specificity, accuracy, and AUROC of our proposed network architecture achieved the highest performance compared to the others mentioned above. Furthermore, the total number of parameters used in our proposed model was 560,000, which was relatively fewer parameters than that used with AlexNet (83.45 times larger) and Sketch-a-Net (14.96 times larger). Although the total number of parameters in our previous work was 0.4 million (1.4 times smaller), the accuracy and AUROC of our proposed model increased by 6.6% and 6.2%, respectively. Moreover, it took only 2 minutes to complete 1-fold training of our proposed network architecture compared to AlexNet (10 minutes), Sketch-a-Net (29 minutes), and our previous work (9 minutes). Figure 7 depicts the ROC curves of the different classifiers.

### Effectiveness of the Transfer Learning Technique

A comparison of models with or without use of the transfer learning strategy was carried out using the images drawn by the participants in the delayed recall trial. In order to validate the effectiveness of the transfer learning method, the network applied the same structure as that of the convolutional base of the pretraining model using the TU-Berlin sketch data set mentioned in the Methods section. The network was composed of five 3×3 convolutional layers; a 2×2 max pooling layer followed each convolution layer, and two FC layers with neurons were used to discriminate the images drawn by the participants. Moreover, the dropout and data augmentation techniques were also implemented. In addition, we compared the transfer learning technique using Sketch-a-Net’s network architecture [37]. First, the network architecture of Sketch-a-Net was pretrained using the TU-Berlin data set [36]. The pretrained model was then transferred to our NTUH_ROCF data set, and data augmentation was also implemented for further training and classification of participants with MCI or HCs.

As a result, the transfer learning technique, which pretrained using a larger data set, achieved better performance, with a mean sensitivity of 0.847 (SD 0.017), a mean specificity of 0.905 (SD 0.009), a mean accuracy of 0.876 (SD 0.010), and a mean AUROC of 0.913 (SD 0.004). When the transfer learning technique was not used, the model performance achieved a mean sensitivity of 0.749 (SD 0.030), a mean specificity of 0.814 (SD 0.017), a mean accuracy of 0.781 (SD 0.014), and a mean AUROC of 0.846 (SD 0.005), as shown in Table 7. Moreover, our proposed network achieved better results than the Sketch-a-Net with the transfer learning architecture.

**Table 7:** Performance of network architectures with and without transfer learning applied to the NTUH_ROCF^a^ data set.

Metric	Sketch-a-Net: with transfer learning, mean (SD)	Our proposed model, mean (SD)	
		Without transfer learning	With transfer learning
Sensitivity	0.641 (0.040)	0.749 (0.030)	0.847 (0.017)
Specificity	0.810 (0.022)	0.814 (0.017)	0.905 (0.009)
Accuracy	0.725 (0.010)	0.781 (0.014)	0.876 (0.010)
AUROC^b^	0.819 (0.010)	0.846 (0.005)	0.913 (0.004)

^a^NTUH_ROCF: National Taiwan University Hospital_Rey-Osterrieth Complex Figure.

^b^AUROC: area under the receiver operating characteristic curve.

## Discussion

### System Usage

The developed system is applicable for use as an early-stage screening system in hospitals. It could help clinicians diagnose patients with MCI and AD. It could also help clinicians assess patients’ visual perception and their ability to retrieve learned information, in order to test their long-term visual memory function. The accuracy, reliability, and efficiency of the screening system is important for diagnosing patients correctly.

### Limitations of This Study

For the data set, as ground truth, it is assumed that the participants were diagnosed correctly by experienced doctors and neuropsychologists. To study the designed system that has been proposed, only the characteristics that are detectable from the neuropsychology test were involved. Therefore, it is a challenge to have participants participate in the study. For research purposes, the number of data sets obtained was minimal, and the data set was only collected locally in Taiwan. Therefore, the data set is biased. As for this study’s research purpose, the study was focused on distinguishing participants with AD and MCI from HCs in an Asian older adult population. In order to obtain more generalized data sets to reduce overfitting, further data need to be collected from participants of different ethnicities and age groups. This system is only useful for one specific neuropsychological test: the ROFC test. In the future, incorporation with other neuropsychological tests will improve the performance of the screening system.

### Conclusions

For decades, AD has been one of the most common diseases among older adults. It is challenging to identify the difference in cognitive performance between patients with MCI and people experiencing normal aging, as the difference may be very subtle, particularly at the early stage of MCI. Nevertheless, early identification of individuals with a high risk of developing AD will help in the management and support of the long-term quality of life of patients with AD and their caregivers. Neuropsychology and cognitive ability can be tested during the screening process, and they do not require any sophisticated medical equipment. Among different types of cognitive testing, clinicians and neuropsychologists often use the ROCF test to help with diagnosing patients. However, it involves intensive labor, and the tester must be qualified as an expert. Data-driven deep learning approaches, which can extract features automatically, have opened the door to the possibility of assisting clinicians, such as neurologists, and clinical neuropsychologists during screening by making the diagnosis process more effective than the traditional approach. With the aid of transfer learning and deep learning, we have proposed an automatic digital screening system to characterize hand-drawn images. It allows us to effectively distinguish patients with MCI and AD from people experiencing normal aging based on the ROCF test process.

The digital screening system that was developed in this study has shown promising preliminary results regarding distinguishing patients with AD and MCI from HCs. Therefore, this screening system can be used during early assessments to diagnose individuals with a high risk of AD. The results have also shown that the system performed better when distinguishing patients with AD from HCs, since there is a significant characteristic difference, as compared to distinguishing patients with MCI from HCs. After analyzing the drawn images, the scores were calculated automatically, and the calculation time was swift. Therefore, this system can replace the labor-intensive and time-consuming work that comes with manually calculating scores according to the criteria of the scoring system. For future studies, merging additional data from various types and stages of dementia will increase the capability of our system in assisting clinicians. Moreover, other types of neuropsychological tests can be included through ensemble methods to provide a complete screening system.

## Abbreviations

AD: Alzheimer disease
AdaGrad: adaptive gradient algorithm
AUROC: area under the receiver operating characteristic curve
BQSS: Boston Qualitative Scoring System
CDT: Clock-Drawing Test
CNN: convolutional neural network
D: Alzheimer disease, in the context of NTUH_D-ROCF
FC: fully connected
FN: false negative
FP: false positive
HC: healthy control
IRB: Institutional Review Board
MCI: mild cognitive impairment
MMSE: Mini-Mental State Examination
MRI: magnetic resonance imaging
NTUH: National Taiwan University Hospital
PET: positron emission tomography
ReLU: rectified linear unit
RMSProp: root mean square propagation
ROC: receiver operating characteristic
ROCF: Rey-Osterrieth Complex Figure
SVM: support vector machine
TN: true negative
TP: true positive
TU: Technical University
